# MEKK5 Interacts with and Negatively Regulates the E3 Ubiquitin Ligase NEDD4 for Mediating Lung Cancer Cell Migration

**DOI:** 10.3390/life11111153

**Published:** 2021-10-29

**Authors:** Aiqin Sun, Jun Zhu, Song Xia, Yanling Li, Tiantian Wu, Genbao Shao, Wannian Yang, Qiong Lin

**Affiliations:** School of Medicine, Jiangsu University, Zhenjiang 212013, China; aiqinsun@ujs.edu.cn (A.S.); 2111913020@stmail.ujs.edu.cn (J.Z.); 2211913081@stmail.ujs.edu.cn (S.X.); 2211913003@stmail.ujs.edu.cn (Y.L.); 2212013086@stmail.ujs.edu.cn (T.W.); gbshao07@ujs.edu.cn (G.S.)

**Keywords:** MEKK5, NEDD4, E3 ubiquitin ligase, ubiquitination, cell migration

## Abstract

Our previous studies have shown that the HECT E3 ubiquitin ligase NEDD4 and kinase MEKK5 both play an essential role in lung cancer migration. A report predicts that MEKK5 may be ubiquitinated by NEDD4; however, interaction of MEKK5 with NEDD4 and ubiquitination of MEKK5 by NEDD4 have not been characterized. In this report, we show that NEDD4 interacts with MEKK5 through a conserved WW3 domain by the co-immunoprecipitation and the GST-pulldown assays. The ubiquitination assay indicates that MEKK5 is not a ubiquitination substrate of NEDD4, but negatively regulates NEDD4-mediated ubiquitination. Furthermore, overexpression of MEKK5 significantly reduced the NEDD4-promoted lung cancer cell migration. Taken together, our studies have defined an inhibitory role of MEKK5 in regulation of NEDD4-mediated ubiquitination.

## 1. Introduction

Neuronal precursor cell-expressed developmentally downregulated 4 (NEDD4), a well-known HECT domain-containing E3 ubiquitin ligase, was originally identified in the early embryonic mouse central nervous system and plays important roles in numerous cellular processes by interacting with many proteins [1,2,3,4]. Early studies showed that NEDD4 ubiquitinates the epithelial sodium channel (ENaC) for proteasomal degradation and thus modulates ENaC abundance [5,6]. Subsequent studies broadened the functional spectrum of NEDD4 and found that NEDD4 is involved in regulating receptor endocytic trafficking, viral budding, and the neural development process [7,8,9,10,11]. Furthermore, our recent studies observed that NEDD4 directly interacts with the key autophagosomal protein LC3B via its LC3-interactive region (LIR) and ubiquitinates the autophagic receptor SQSTM1, suggesting that NEDD4 may function in selective autophagy [12,13]. In addition, accumulated evidence indicates that NEDD4 expression is frequently upregulated in several human malignancies and is associated with tumorigenesis and progression [14,15,16,17]. Our previous studies found that aberrant overexpression of NEDD4 in gastric cardia adenocarcinoma is correlated with the tumor invasion and metastasis [18] and NEDD4 mediates the EGFR lung cancer cell migration signaling through interacting with the EGFR signaling complex and promoting lysosomal secretion of cathepsin B [19]. However, how the E3 ubiquitin ligase activity of NEDD4 is regulated in cells remains elusive.

MEKK5, known as apoptosis signal-regulating kinase 1 (ASK1), is a serine/threonine kinase belonging to the MAP3K family [20]. MEKK5 is ubiquitously expressed and involved in regulation of the JNK and p38 pathways in response to various cytotoxic stresses [21,22,23]. Previous studies have shown that MEKK5 plays essential roles in the pathogenesis of various diseases, including inflammatory diseases, cardiovascular diseases, neurodegenerative diseases, and cancers [24,25,26,27,28,29]. Our recent study has demonstrated MEKK5 inhibits lung cancer cell proliferation and migration through interacting with and inactivating transcriptional coactivator with PDZ-binding motif (TAZ) [30].

We showed previously that either knockdown of NEDD4 or overexpression of MEKK5 significantly reduces lung cancer cell migration [19,30]. In addition, MEKK5 contains a PPFY motif and has been predicted to be a ubiquitination substrate of NEDD4 [31]. However, physical interaction and functional connection between NEDD4 and MEKK5 have not been characterized. In this research article, we found that NEDD4 interacts with MEKK5 via its WW domain. The ubiquitination assay indicates that MEKK5 is involved in regulation of the E3 ubiquitin ligase activity of NEDD4 but is not a ubiquitination substrate of NEDD4. Furthermore, overexpression of MEKK5 inhibits the NEDD4-mediated lung cancer cell migration.

## 2. Materials and Methods

### 2.1. Reagents

Antibodies: anti-MEKK5 (F-9; SC-5294) and anti-ACK (A11; SC-28336) were purchased from Santa Cruz Biotechnology (Dallas, TX, USA), anti-NEDD4 (07–049) was from Millipore (Billerica, MA, USA), anti-GFP (MMS-118R) and anti-ubiquitin (P4G7; MMS-258R) were from BioLegend (San Diego, CA, USA), anti-Myc (MMS-150R) and anti-HA (MMS-101R) were from Covance (Princeton, NJ, USA), anti-tubulin (BS1699) was from Bioworld Technology (Shanghai, China), and anti-actin (A5441) was from Sigma-Aldrich (St. Louis, MO, USA). Protein A–Sepharose beads (P3391) and glutathione agarose (G-4510) were purchased from Sigma-Aldrich (St. Louis, MO, USA). Secondary antibodies were HRP-conjugated goat anti-mouse (31430) and goat anti-rabbit IgG (31460) from ThermoFisher Scientific (Waltham, MA, USA). MG-132 was purchased from Sigma-Aldrich (St. Louis, MO, USA). The MEKK5 and luciferase (control) shRNA oligos were synthesized by Sangon Biotech (Shanghai, China) Company. The lung cancer cell lines A549 and NCI-H1650 were purchased from the American Type Culture Collection (ATCC).

### 2.2. Cell Culture and Transfection

HEK293T, NCI-H1650, and A549 cells were cultured in DMEM with 10% fetal bovine serum, 100 units/mL p/s at 37 °C with 5% CO_2_. Transfections of the plasmid DNAs were performed using transfection reagent Transfecgen (Newgen Biotech, Zhenjiang, China) according to the manufacturer’s instructions.

### 2.3. Construction of Plasmids and Mutagenesis

The mammalian expression plasmids pcDNA3, pcDNA3-HA-NEDD4, pcDNA3-HA-NEDD4-2, pcDNA3-Myc-ACK1, GFP-SQSTM1, and pGEX4T3-ACK1-UBA were previously described [13,32]. Human SCYL1 or its mutant cDNA was subcloned into the lentiviral expression vector FUW-Myc for establishing stable cell lines in A549 cells and into the mammalian expression vector pcDNA3-Myc for transient transfection in HEK293T cells. Point mutations were generated by site-directed mutagenesis PCR and overlapping PCR using pcDNA3-Myc-MEKK5 as the template. The MEKK5 shRNAs were subcloned into the lentiviral shRNA expression vector pLKO.1 (Addgene) via AgeI/EcoRI sites. The constructs were verified by DNA sequencing.

### 2.4. Virus Packaging and Transduction

The viral packaging was performed as described previously [13,33]. Briefly, the lentiviral plasmids were transfected into HEK293T cells together with a packaging plasmid psPAX2 (Addgene) and envelope plasmid pMD2.G (Addgene) using Transfecgen transfection reagent. The culture medium was changed to remove the transfection reagent for 12 h after the transfection. The lentivirus-containing culture medium was harvested every 24 h for 3 times and centrifuged at 1250 rpm for 5 min or passed through a 0.45 µm filter to remove any HEK293T cell debris. The target cells were infected with 0.5 mL lentiviral particles in the presence of 6 μg/mL polybrene (Sigma-Aldrich, St. Louis, MO, USA), and puromycin (Biotopped, Beijing, China) was used to select the infected cells.

### 2.5. Immunoprecipitation and Immunoblotting

In immunoprecipitation experiments, cells were lysed 48 h after transfection in precooled mammalian cell lysis buffer by rocking plates at 4 °C for 30 min. Whole-cell lysates were centrifuged at 12,000 rpm for 15 min at 4 °C to remove the insoluble fraction. The precleared cell lysate was incubated with primary antibody on ice for 30 min, and then 20 μL of the 50% slurry protein A–Sepharose beads was added, and the mixture was incubated with rotation at 4 °C for 3 h. The immunoprecipitates were washed 4 times with lysis buffer. The SDS-PAGE lysate samples were prepared by addition of 5× SDS sample buffer directly to the whole-cell lysates or immunoprecipitated proteins and boiled at 100 °C for 5–10 min, resolved by 8–12% SDS-PAGE. The separated proteins were transferred from the gel to PVDF membranes (Millipore); blots were treated with primary antibody overnight at 4°C and subsequently with a secondary antibody for 2 h at room temperature. Detection was performed with the Western Lightning Immunoblot Kit (Beyotime, Shanghai, China).

### 2.6. Pulldown Assays with GST Fusion Protein

This assay was carried out as previously described [32]. GST fusion proteins were expressed in DH5α and purified via affinity purification with glutathione agarose beads. The glutathione agarose bead bound GST fusion proteins (20–30 μg) were incubated with the whole-cell lysates (~1 mg) at 4 °C for 3 h, followed by washing with the mammalian cell lysis buffer 4 times. Following the SDS-PAGE lysate samples were prepared by adding 5× SDS sample buffer directly to the washed beads and lysates. After electrophoresis, the separated proteins were transferred to a PVDF membrane for immunoblotting.

### 2.7. Detection of Ubiquitinated Proteins

Detection of ubiquitinated proteins was performed using both GST-UBA pulldown and immunoprecipitation assays as described previously [12]. For GST-UBA pulldown assay, the ubiquitinated proteins in the lysates were precipitated by GST-UBA-conjugated beads, and the ubiquitinated protein was detected by immunoblotting with an anti-GFP or anti-ACK1 antibody. For immunoprecipitation assay, ubiquitinated proteins were immunoprecipitated by primary antibody and immunoblotted with an anti-ubiquitin antibody.

### 2.8. In Vitro Ubiquitination Assay

To examine in vitro ubiquitination of target substrate protein by NEDD4, the target plasmid was transfected into HEK293T cells and immunoprecipitated with specific primary antibody and protein A beads. In vitro ubiquitination of substrate protein was performed by adding 15 µL reaction mixture (100 nM E1, 0.5 µM UbcH7 (E2), 5 µM monoubiquitin, and 2 mM ATP in ULR buffer (25 mM Tris-HCl [pH 8.0], 100 mM NaCl, 2.0 mM MgCl2, and 1 mM dithiothreitol)) to 10 µL of the ligase/substrate fraction at 22 °C for 30 min, followed by washing with mammalian cell lysis buffer 3 times to remove nonsubstrate protein-conjugated ubiquitin, and resuspended in 6.25 μL 5× SDS-PAGE sample buffer for electrophoresis.

### 2.9. Cell Migration Assays

The ability of cell migration was determined by the transwell assay. Transwell migration assay was performed in transwell chambers (Corning, NY, USA). Cells (4 × 10^4^) resuspended in 200 μL serum-free DMEM were plated onto the top of each chamber insert. DMEM medium containing 10% FBS was added to the bottom of the chamber. After incubation at 37 °C for an indicated time, cells on the upper surface were gently removed with a cotton swab. Cells that migrated into the bottom of the membrane were fixed with 3.7% paraformaldehyde for 30 min and stained with Crystal Violet Staining Solution (Beyotime, Shanghai, China) for 10 min. The stained cells were washed with PBS 3 times and then imaged under a phase microscope. The stained cell numbers were counted in five random fields of view.

### 2.10. Statistical Analysis

Three experiments at minimum were completed for each analysis. Data are presented as mean ± standard deviation. Statistical difference was analyzed using Student’s t-test. P value less than 0.05 was considered statistically significant.

## 3. Results

### 3.1. MEKK5 Specifically Interacts with NEDD4

**Figure 1:**
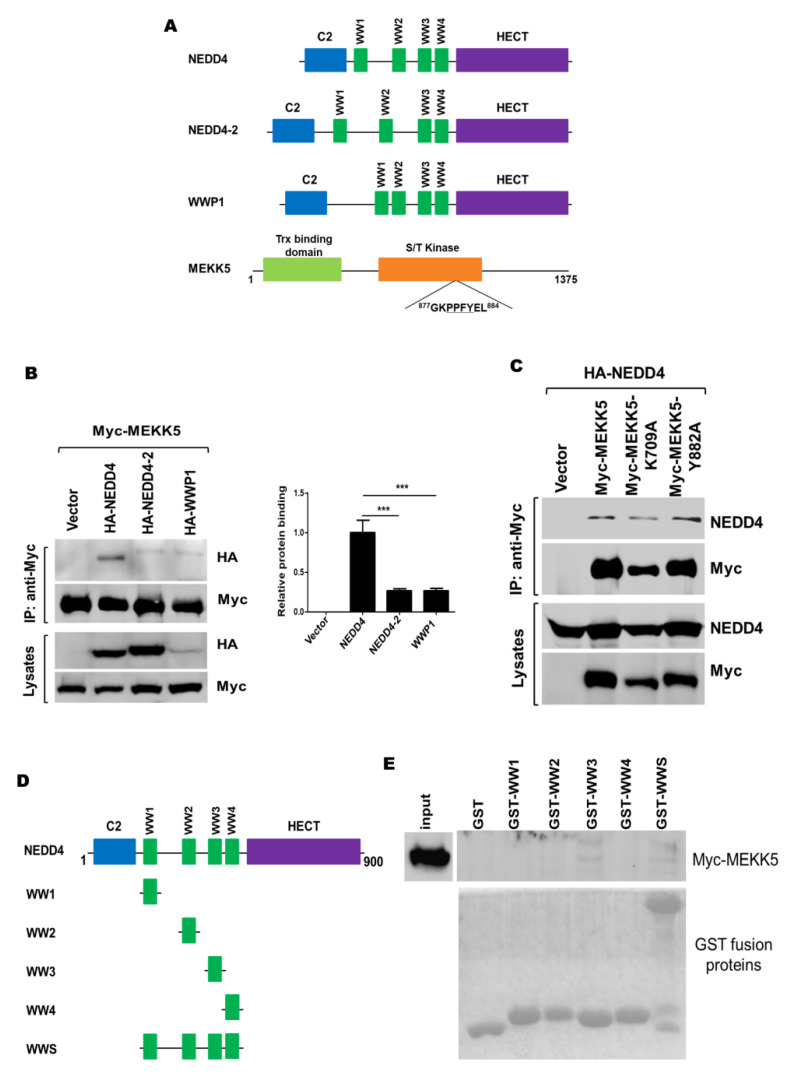
MEKK5 specifically interacts with NEDD4. (**A**) Domain structure of three NEDD4 family E3 ubiquitin ligases and MEKK5. (**B**) HA-NEDD4, NEDD4-2, WWP1, or vector was coexpressed with Myc-MEKK5 in HEK293T cells. Myc-MEKK5 was immunoprecipitated with anti-Myc antibody; coprecipitated proteins were detected by immunoblotting with anti-HA antibody. (**C**) NEDD4 was cotransfected with Myc-MEKK5 or the mutants into HEK293T cells. Myc-MEKK5 or the mutants were immunoprecipitated with an anti-Myc antibody, and co-immunoprecipitated HA-NEDD4 was detected by immunoblotting with an anti-NEDD4 antibody. (**D**) Schematic representation of GST-NEDD4 WW domain constructs. (**E**) The bead-bound GST or GST-NEDD4-WW domain was incubated with the Myc-MEKK5-expressed HEK293T cell lysates. The coprecipitated MEKK5 was detected by immunoblotting with an anti-Myc antibody. *** *p* < 0.001.

It is known that NEDD4 family E3 ubiquitin ligases have a central region containing 2–4 WW domains for interaction with the PPXY motif or the phospho-serine/threonine residues in substrates (Figure 1A) [34,35]. MEKK5 was identified to be a putative ubiquitination substrate of NEDD4 by an in vitro substrate screening assay [31]. Sequence analysis showed that MEKK5 has a PPFY motif, suggesting that NEDD4 family E3 ubiquitin ligases may directly interact with MEKK5. To identify which NEDD4 family E3 ligase was the preferential binding protein for MEKK5, we coexpressed MEKK5 with NEDD4, NEDD4-2, and WWP1 and detected the interaction between MEKK5 and these three E3 ligases by co-immunoprecipitation in the HEK293T cells. As shown in Figure 1B and Figure S1, all the three ligases are capable of binding to MEKK5, whereas NEDD4 bound to MEKK5 with a much higher affinity. To determine whether the ^879^PPFY^882^ is the site interacting with NEDD4, we mutated Y882 into alanine in MEKK5 and tested the binding of the mutant to NEDD4 using a co-immunoprecipitation assay. To our surprise, the mutant MEKK5-Y882A was able to coprecipitate NEDD4 from the lysates to the same extent as wild-type MEKK5 (Figure 1C and Figure S1), demonstrating that the ^879^PPFY^882^ is not the site for MEKK5 to bind to NEDD4. In addition, we observed that the kinase-dead (KD) mutant MEKK5-K709A did not significantly affect the binding to NEDD4.

To identify the region in NEDD4 that interacts with MEKK5, we made a series of GST-NEDD4 WW domain constructs (Figure 1D) and performed the GST fusion protein pulldown assay. As shown in Figure 1E and Figure S1, the bead-bound GST-NEDD4-WW3 and GST-NEDD4-WWS precipitated Myc-MEKK5 from the cell lysates, while the GST-NEDD4-WW1, GST-NEDD4-WW2, and GST-NEDD4-WW4 could not, indicating that NEDD4 binds to MEKK5 through the WW3 domain.

### 3.2. MEKK5 Is Not a Ubiquitination Substrate of NEDD4

**Figure 2:**
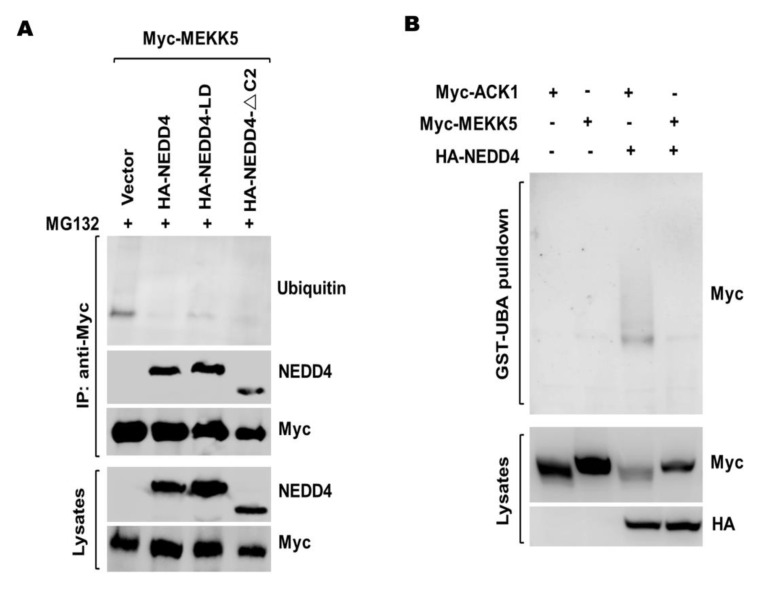
MEKK5 is not a ubiquitination substrate of NEDD4. (**A**) Myc-MEKK5 and NEDD4 or its mutants were coexpressed in HEK293T cells, Treatment with MG-132 (10 μM) was carried out 18 h before harvesting the cells. Myc-MEKK5 was immunoprecipitated by anti-Myc antibody and immunoblotted with an anti-ubiquitin antibody. (**B**) NEDD4 was cotransfected with Myc-MEKK5 or Myc-ACK1 into HEK293T cells; ubiquitinated proteins were precipitated by GST-UBA and immunoblotted with an anti-Myc.

To verify if MEKK5 is a ubiquitination substrate of NEDD4, we performed the immunoprecipitation assay. The Myc-tagged MEKK5 was ectopically expressed alone or coexpressed with HA-NEDD4 in HEK293T cells. Ubiquitination of MEKK5 was determined by anti-Myc IP followed by immunoblot with an anti-ubiquitin antibody. As shown in Figure 2A and Figure S2, MEKK5 showed no increase in ubiquitination when coexpressed with NEDD4, and both the ligase-dead (LD) mutant NEDD4-C867A and the N-terminal C2 domain truncation mutant NEDD4-△C2 did not affect the ubiquitination of MEKK5. To confirm this result, we performed the GST-UBA pulldown assay to detect the ubiquitination level of MEKK5 upon coexpression with NEDD4 in HEK293T cells. As shown in Figure 2B and Figure S2, ubiquitination of MEKK5 was barely detectable in the presence of NEDD4, while Myc-ACK1, which is a known ubiquitination substrate of NEDD4 [32], was ubiquitinated when coexpressed with NEDD4. These results indicate that MEKK5 may not be a ubiquitination substrate of NEDD4 in cells.

### 3.3. MEKK5 Inhibits the Ubiquitination Activity of NEDD4

**Figure 3:**
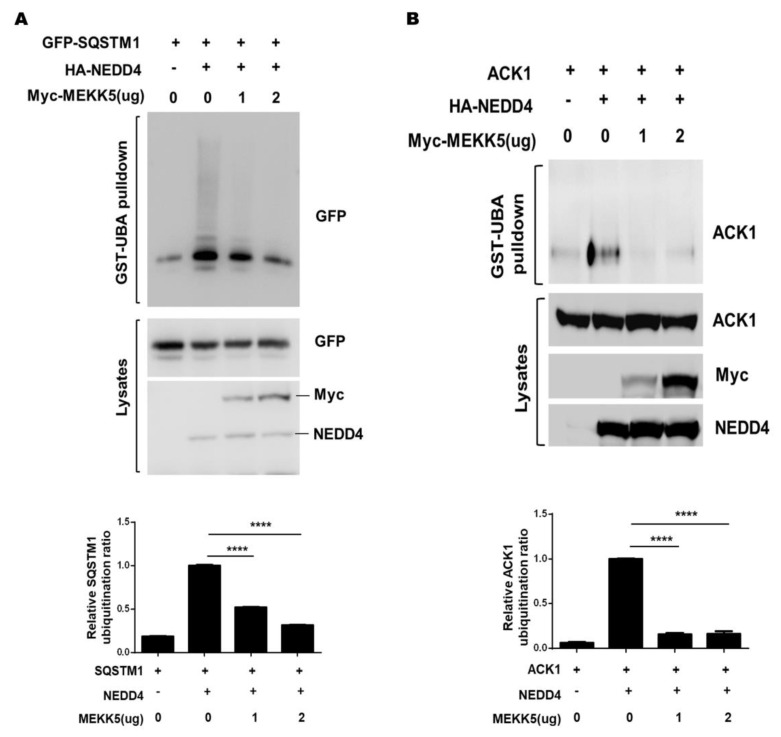
MEKK5 inhibits ubiquitination of substrates by NEDD4 in vivo. (**A**,**B**) GFP-SQSTM1 or ACK1 and HA-NEDD4 were transfected into HEK293T cells with Myc-MEKK5 or empty vectors. The ubiquitinated SQSTM1 or ACK1 was precipitated with GST-UBA and detected by immunoblotting with anti-GFP or anti-ACK1. **** *p* < 0.0001.

Because our studies did not observe that NEDD4 ubiquitinates MEKK5, we wonder if the interaction of MEKK5 with NEDD4 might be involved in the regulation of the NEDD4-mediated ubiquitination. SQSTM1 and ACK1 are the substrates of NEDD4 [12,32]. We used the GST-UBA pulldown assay to determine the NEDD4-dependent ubiquitination level of SQSTM1 and ACK1 upon coexpression with Myc-MEKK5. As shown in Figure 3A,B and Figure S3, both SQSTM1 and ACK1 were heavily ubiquitinated by NEDD4. However, when coexpressed with MEKK5, the ubiquitination of SQSTM1 and ACK1 by NEDD4 was significantly reduced. These results suggest MEKK5 inhibits the E3 ligase activity of NEDD4.

**Figure 4:**
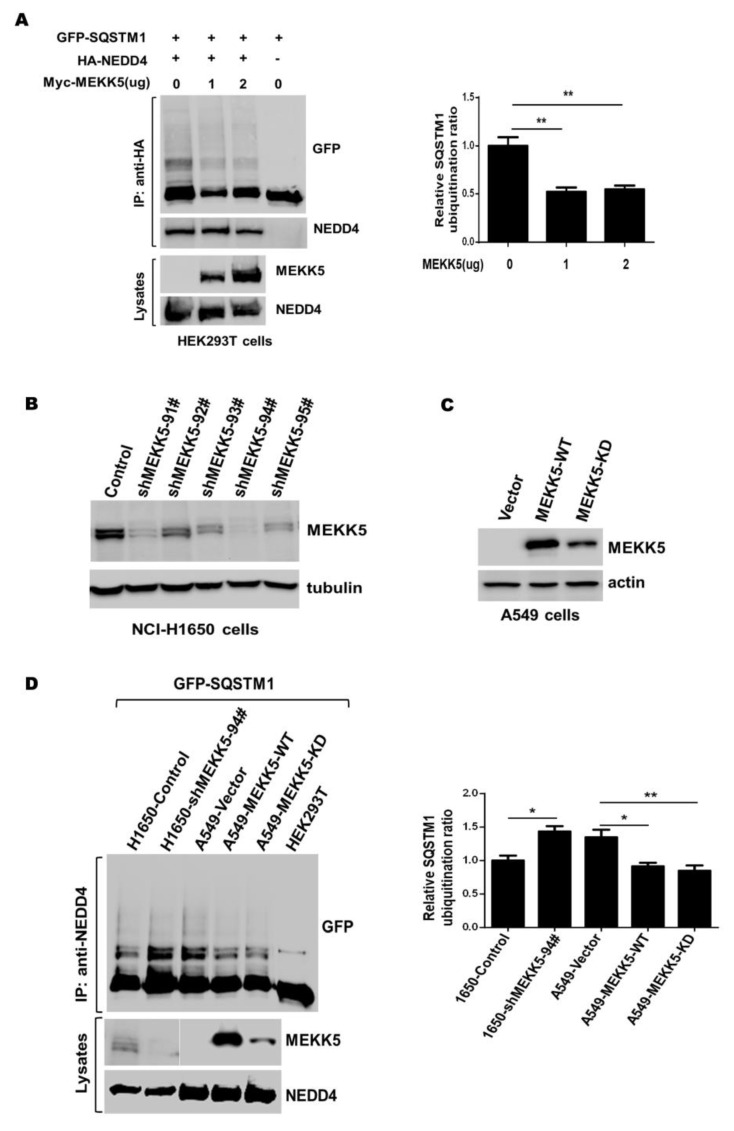
MEKK5 inhibits ubiquitination of substrates by NEDD4 in vitro. (**A**) Myc-MEKK5 was cotransfected with HA-NEDD4 or the vector into HEK293T cells, immunoprecipitated with an anti-HA antibody and added indicated amount of purified GFP-SQSTM1 protein, and used for the in vitro ubiquitination assay. The ubiquitination of SQSTM1 was detected by immunoblotting with anti-GFP antibody. (**B**) The lung cancer NCI-H1650 cells were infected by lentiviral vector-loaded MEKK5 shRNA for 24 hours, and endogenous MEKK5 in the cell lysates was detected by immunoblotting with anti-MEKK5. (**C**) The MEKK5 (MEKK5-WT) or its kinase-dead mutant MEKK5-K709A (MEKK5-KD) was stably expressed in the lung cancer A549 cells using a lentiviral mammalian expression system. The expressed protein level of MEKK5 or its kinase-dead mutant was detected by immunoblotting of the cell lysates with anti-MEKK5. (**D**) MEKK5 knockdown and overexpression cell lines established in lung cancer NCI-H1650 and A549 cells were immunoprecipitated with an anti-MEKK5 antibody. The indicated amount of purified GFP-SQSTM1 protein was added into immunoprecipitated proteins before the in vitro ubiquitination assay. Ubiquitination of SQSTM1 was detected by immunoblotting with an anti-GFP antibody. * *p* < 0.05, ** *p* < 0.01.

We further examined the effect of MEKK5 on the NEDD4-mediated ubiquitination of SQSTM1 using the in vitro ubiquitination assay. The same inhibitory effect of MEKK5 on the NEDD4-mediated ubiquitination was observed (Figure 4A and Figure S4). In order to confirm the results, we established the MEKK5 knockdown cell lines in lung cancer NCI-H1650 cells and the MEKK5 overexpression cell lines in lung cancer A549 cells using a lentiviral expression system, and we detected the knockdown or the overexpression effect on the MEKK5 protein level. The Western blot revealed that both shMEKK5-91# and shMEKK5-94# effectively reduced the MEKK5 protein level in NCI-H1650 cells, while overexpression of MEKK5 markedly increased the MEKK5 protein level in A549 cells (Figure 4B,C and Figure S4). We next performed in vitro ubiquitination assay to determine the effect of the knockdown or the overexpression of MEKK5 on the NEDD4-mediated ubiquitination. Compared with the vector control cell line, knockdown of MEKK5 significantly enhanced the ubiquitination of SQSTM1 by NEDD4, while overexpression of MEKK5 dramatically reduced the ubiquitination level of SQSTM1 (Figure 4D and Figure S4). Taken together, these results suggest an inhibitory role of MEKK5 in regulating the E3 ligase activity of NEDD4.

### 3.4. The Inhibitory Effect of MEKK5 on Cell Migration Might Be Produced from Negative Regulation of NEDD4

**Figure 5:**
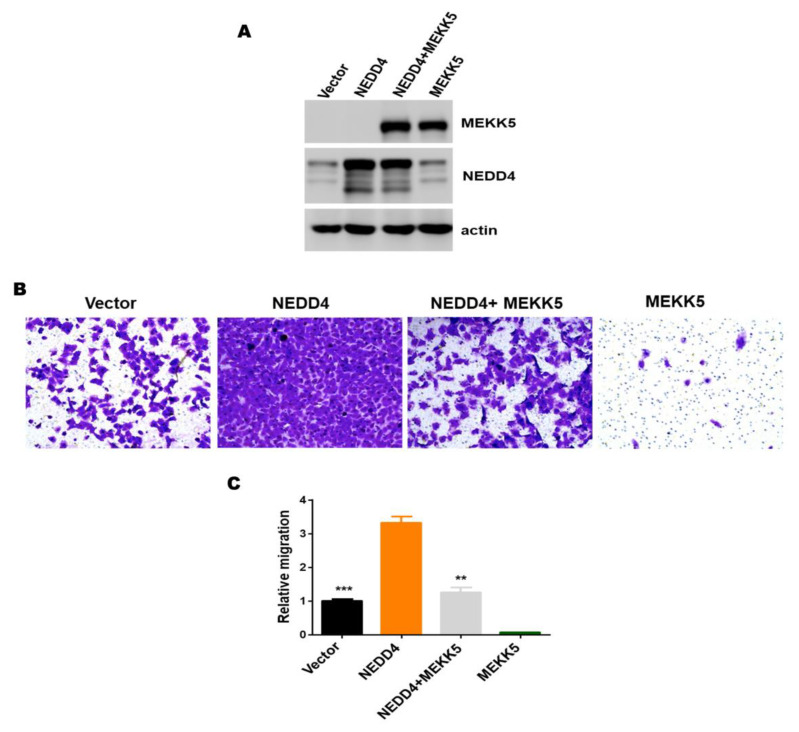
Overexpression of MEKK5 produces an inhibitory effect in the NEDD4 migration signaling. (**A**) Overexpression of NEDD4 and MEKK5 upon introducing NEDD4 and MEKK5 cDNA in the cells. (**B**,**C**) The effects of overexpression of NEDD4 and MEKK5 on cell migration were determined by the transwell assay. For quantification in the transwell assay, the migrated cells were fixed, stained with crystal violet, and counted under a microscope from three randomly selected fields. ** *p* < 0.01, *** *p* < 0.001.

To investigate the biological role of the interaction of MEKK5 with NEDD4, we first made a stable NEDD4 and MEKK5 overexpression cell line in lung cancer A549 cells separately (Figure 5A and Figure S5) and carried out a transwell assay. In agreement with our previous findings [19], overexpression of NEDD4 significantly promoted the migration of A549 cells, and overexpression of MEKK5 inhibited cell migration (Figure 5B,C). We subsequently examined the effect of MEKK5 overexpression on the NEDD4-mediated lung cancer cell migration signaling. As shown in Figure 5B,C, overexpression of MEKK5 in the NEDD4 overexpressed cells eliminated NEDD4-promoted cell migration capacity. The above data suggest that overexpression of MEKK5 inhibits the lung cancer cell migration through negative regulation of the NEDD4 E3 ubiquitin ligase activity. Our studies have provided a new regulatory mechanism underlying the NEDD4-promoted cancer cell migration.

## 4. Discussion

The HECT E3 ubiquitin ligase NEDD4 plays an important role in regulating cancer progression [14,15,16,17]. We recently reported that the expression of NEDD4 in gastric cardia adenocarcinoma tissues was significantly higher than that in adjacent normal tissue and was tightly associated with the prognosis of patients and gastric cardia adenocarcinoma metastasis [18]. Knockdown of NEDD4 by shRNA completely impairs cell migration and invasion of gastric cancer cells [18]. Furthermore, we found that NEDD4 promotes lung cancer cell migration by facilitating the EGFR-dependent lysosomal secretion of cathepsin B [19]. In this report, we have shown physical interaction between NEDD4 and MEKK5. Overexpression of MEKK5 reduced the NEDD4-mediated ubiquitination, and knockdown of MEKK5 significantly enhanced the E3 ubiquitin ligase activity of NEDD4. More importantly, overexpression of MEKK5 produced an opposite effect on cell migration to that of NEDD4, pointing to an important role of MEKK5 in negatively regulating lung cancer cell migration, probably through inhibiting the NEDD4 ligase activity. These studies have proposed a novel regulatory mechanism underlying the NEDD4-mediated cancer cell migration.

WW domains are small protein modules of 38–40 amino acids that have two conserved tryptophan residues, which play crucial roles in the domain structure and function [36,37,38]. Owing to their intrinsic protein–protein interacting capability, WW domains of NEDD4 family E3 ubiquitin ligases mainly serve to interact with substrate proteins that contain the PPXY motif and other proline-rich motifs [1,39,40]. We demonstrated that NEDD4 physically interacts with MEKK5 through the WW3 domain (Figure 1B,E). Surprisingly, the PPFY sequence in MEKK5 appears not to be the site binding to the WW3 domain of NEDD4 (Figure 1C). Our previous study found MKKK5 also interacts with the WW domains of YAP/TAZ [30]. Currently, we do not know the exact site in MEKK5 that binds to the WW domains. It is necessary for us to identify the WW-interacting site in MEKK5 and make a NEDD4-binding defective mutant of MEKK5 to detect its exact role in regulating the NEDD4-mediated cellular function in future studies.

**Figure 6:**
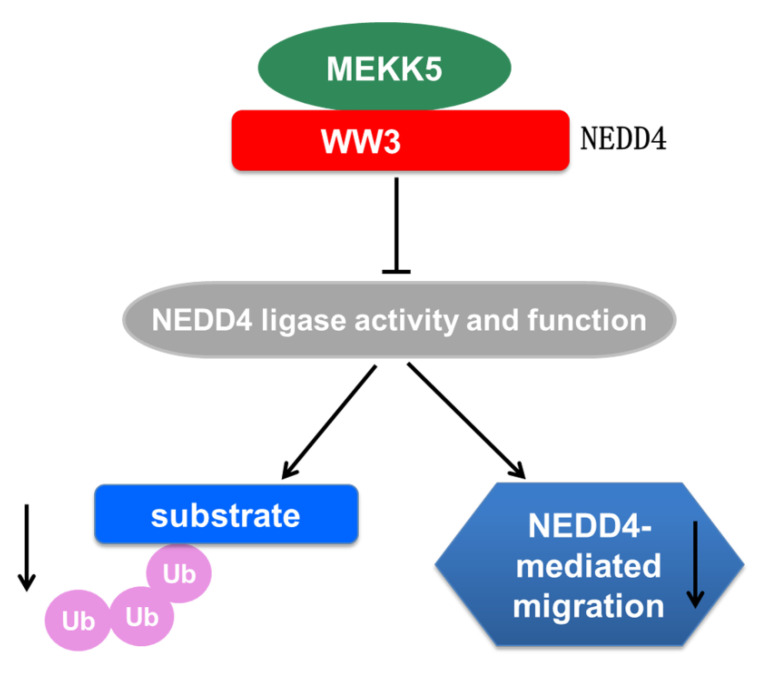
A proposed model of regulation of NEDD4 through interacting with MEKK5. NEDD4 physically interacts with MEKK5 through the WW3 domain. The binding of MEKK5 inhibits the E3 ubiquitin ligase activity of NEDD4, which impairs the NEDD4-mediated ubiquitination and cancer cell migration.

It has been shown that MEKK5 is activated by multiple cellular stress signals and ubiquitination is a major biochemical regulatory means for activation of MEKK5 [21,22,23,41]. To our surprise, MEKK5 showed no increase in ubiquitination when coexpressed with NEDD4 (Figure 2), indicating that MEKK5 was not a ubiquitination substrate of NEDD4. Interestingly, we observed that coexpression of MEKK5 with NEDD4 impaired the NEDD4-mediated ubiquitination of SQSTM1 and ACK1 (Figure 3). Furthermore, we found that knockdown of MEKK5 significantly enhanced the ubiquitination by NEDD4, and overexpression of MEKK5 produced the opposite effect (Figure 4). The results suggest that MEKK5 might be an important negative regulation protein for controlling the NEDD4-mediated ubiquitination. In line with our previous studies, we observed that overexpression of MEKK5 impaired NEDD4-dependent cell migration capacity in lung cancer A549 cells. Our data suggest that the interaction between NEDD4 and MEKK5 efficiently inhibits the NEDD4 migration signaling (Figure 6), which may provide a new strategy for the antimetastatic therapy of lung cancer.

## Data Availability

The data needed to evaluate this work are all included in the manuscript and are available upon request.

## Supplementary Materials

The following are available online at https://www.mdpi.com/article/10.3390/life11111153/s1, Figure S1: MEKK5 specifically interacts with NEDD4—Western blot, Figure S2: MEKK5 is not a ubiquitination substrate of NEDD4—Western blot, Figure S3: MEKK5 inhibits ubiquitination of substrates by NEDD4 in vivo—Western blot, Figure S4: MEKK5 inhibits ubiquitination of substrates by NEDD4 in vitro—Western blot, Figure S5: Overexpression of MEKK5 produces an inhibitory effect in the NEDD4 migration signaling—Western blot.
