# Supplementary material for: MEKK5 Interacts with and Negatively Regulates the E3 Ubiquitin Ligase NEDD4 for Mediating Lung Cancer Cell Migration
Source: Life (Basel). 2021 Oct 29;11(11):1153. doi: 10.3390/life11111153 (PMC8620495; doi:10.3390/life11111153)
Supplement: Supplementary file 1 [file life-11-01153-s001.zip › life-1421805-supplementary.pdf]

Supplementary materials:

MEKK5 interacts with and negatively regulates the E3 ubiquitin ligase NEDD4

Aiqin Sun [1], Jun Zhu [1], Song Xia [1], Yanling Li [1], Tiantian Wu [1], Genbao Shao [1], Wannian Yang [1,*], Qiong Lin [1,*]

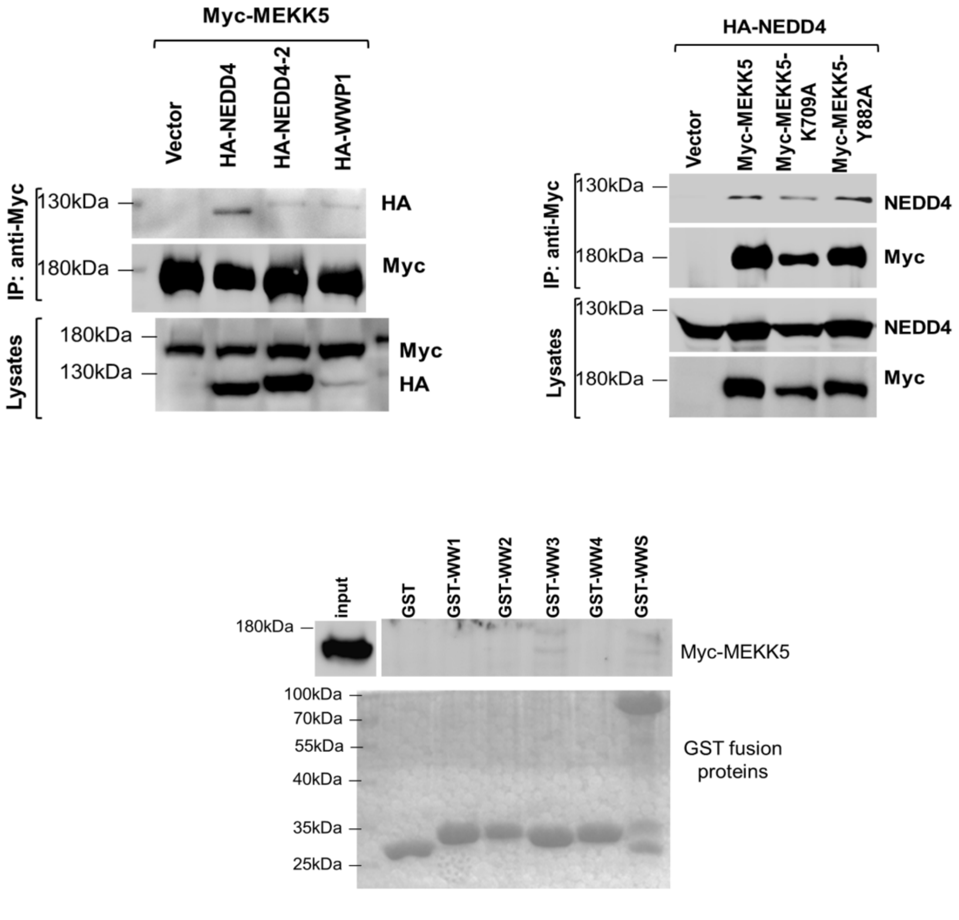

Figure S1. MEKK5 specifically interacts with NEDD4 – western blot

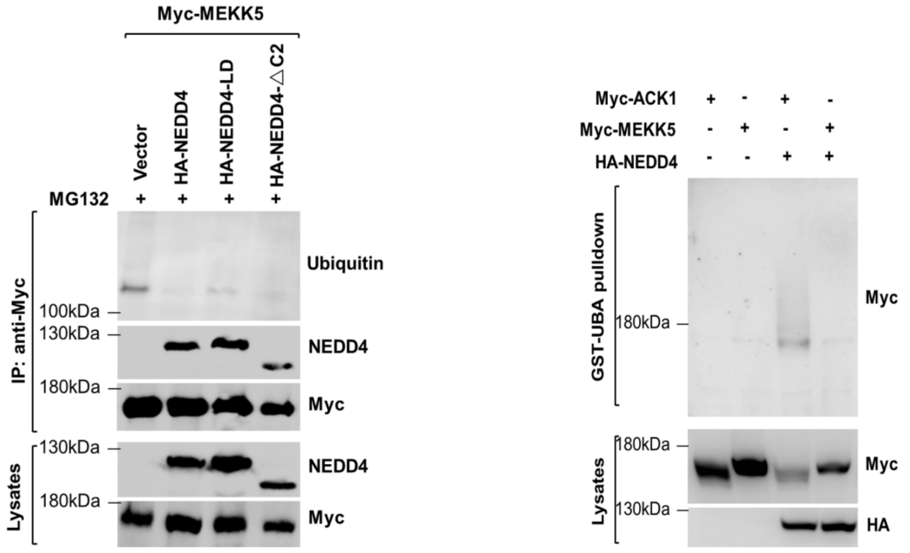

Figure S2. MEKK5 is not an ubiquination substrate of NEDD4 – western blot

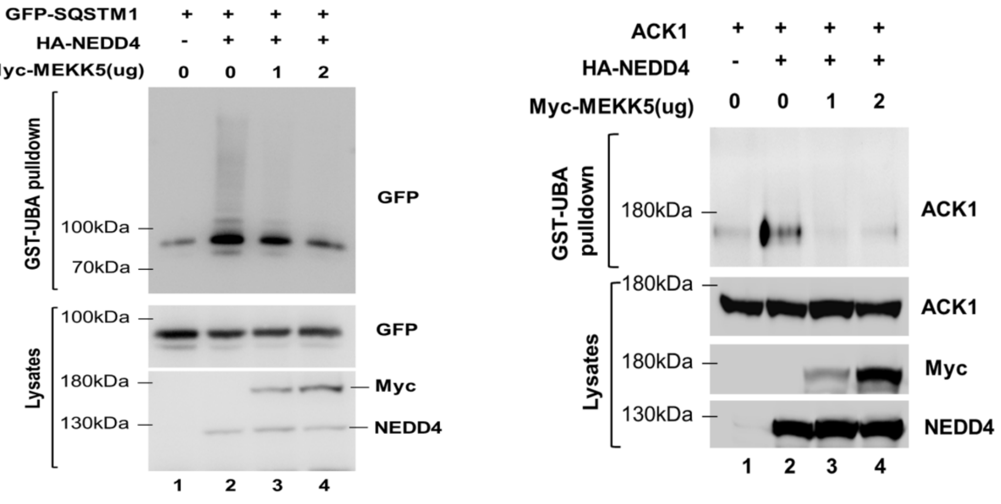

Figure S3. MEKK5 inhibits ubiquitination of substrates by NEDD4 in vivo – western blot

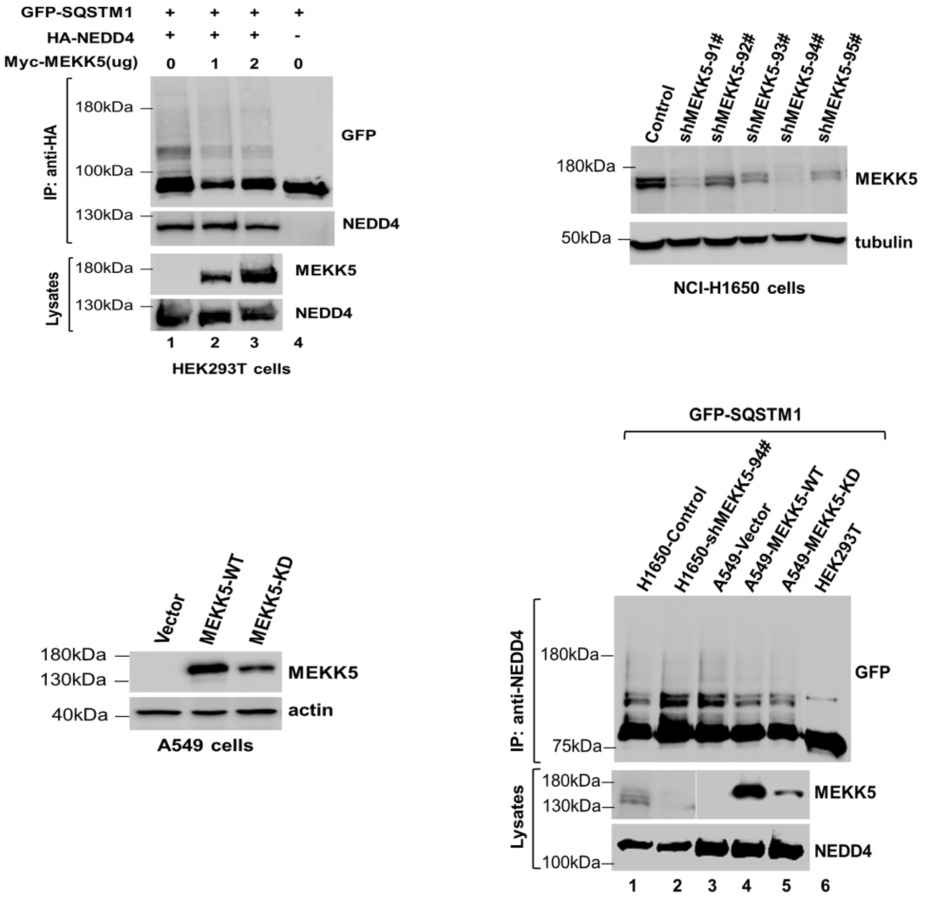

Figure S4. MEKK5 inhibits ubiquitination of substrates by NEDD4 in vitro – western blot

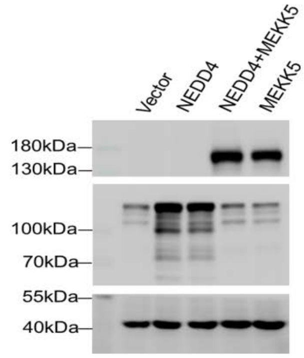

Figure S5. Overexpression of MEKK5 produces an inhibitory effect in the NEDD4 migration signaling – western blot

Table S1. The quantification data of blots

**Figure S1**		Vector				NEDD4				NEDD4-2				WWP1						
	IP:HA	0	0	0		31408	36503	22968		12114	11332	7439		9453	9066	5953				
	IP:Myc	483784	425417	398299		400026	310287	310365		476657	427614	383002		350312	347608	311190				
**Figure S3**		lane1				lane2				lane3				lane4						
	pulldown:GFP	82122	86713	90508		461700	475589	480275		241616	247162	249809		142838	149453	153508				
	pulldown:ACK	0.580222	0.7936	1.050711		13.70756	13.49747	13.76867		1.724356	2.277733	2.432267		1.413811	2.960763	2.185244				
**Figure S4**		lane1				lane2				lane3				lane4				lane5		
	HA IP:GFP	969376	740088	978719		502616	378371	518076		522594	423064	531336								
	NEDD4 IP:GFP	537573	686338	636248		816285	981553	868719		725237	966140	815621		503348	699086	594477		435155	604914	536451
